# Preferred orientation distribution of shock‐induced planar microstructures in quartz and feldspar

**DOI:** 10.1111/maps.13490

**Published:** 2020-06-04

**Authors:** Lidia Pittarello, Ludovic Ferrière, Jean‐Guillaume Feignon, Gordon R. Osinski, Christian Koeberl

**Affiliations:** ^1^ Natural History Museum Vienna Burgring 7 A‐1010 Vienna Austria; ^2^ Department of Lithospheric Research University of Vienna Althanstrasse 14 A‐1090 Vienna Austria; ^3^ Department of Earth Sciences University of Western Ontario 1151 Richmond Street London Ontario Canada N6A 5B7; ^4^ Institute for Earth and Space Exploration University of Western Ontario 1151 Richmond Street London Ontario Canada N6A 3K7

## Abstract

Shocked quartz and feldspar grains commonly exhibit planar microstructures, such as planar fractures, planar deformation features, and possibly microtwins, which are considered to have formed by shock metamorphism. Their orientation and frequency are typically reported to be randomly distributed across a sample. The goal of this study is to investigate whether such microstructures are completely random within a given sample, or whether their orientation might also retain information on the direction of the local shock wave propagation. For this work, we selected samples of shatter cones, which were cut normal to the striated surface and the striation direction, from three impact structures (Keurusselkä, Finland, and Charlevoix and Manicouagan, Canada). These samples show different stages of pre‐impact tectonic deformation. Additionally, we investigated several shocked granite samples, selected at different depths along the drill core recovered during the joint IODP‐ICDP Chicxulub Expedition 364 (Mexico). In this case, thin sections were cut along two orthogonal directions, one parallel and one normal to the drill core axis. All the results refer to optical microscopy and universal‐stage analyses performed on petrographic thin sections. Our results show that such shock‐related microstructures do have a preferred orientation, but also that relating their orientation with the possible shock wave propagation is quite challenging and potentially impossible. This is largely due to the lack of dedicated experiments to provide a key to interpret the observed preferred orientation and to the lack of information on postimpact orientation modifications, especially in the case of the drill core samples.

## Introduction

Paradoxically, the formation of the most studied and used evidence of an impact event, planar deformation features (PDFs) in quartz (e.g., Stöffler and Langenhorst 1994; Grieve et al. 1996; French and Koeberl 2010; and references therein), is still not fully constrained. Different hypotheses of formation have been proposed, including PDFs being traces of glide planes forming to release stress (Engelhardt and Bertsch 1969), PDFs being formed by heterogeneous response of the crystal lattice to shock compression (Grady 1980), PDFs resulting from crystal lattice collapse along specific planes and compensation caused by misfit on both sides of the shock front (Goltrant et al. 1992), and combinations of the previously proposed hypotheses (e.g., Langenhorst 1994; Trepmann 2008). Transmission electron microscopy has revealed that the optically isotropic (apparently amorphous) material in quartz PDFs can be either fully glassy or crystalline, and, in the case of crystalline material, it locally contains microtwins or domains with a high density of dislocations (e.g., Goltrant et al. 1991). Nevertheless, statistical measurements of the abundance of specific crystallographic orientations along which PDFs form in quartz have been proved to be helpful to constrain the shock peak pressure recorded by a given sample (e.g., Grieve et al. [1996] and references therein). Even though the formation of PDFs can be affected by the interaction between shock waves and crystal orientation, the presence of other mineral phases, the porosity, the grain size, etc., shock barometry based on PDF orientations can provide reliable information on shock attenuation with increasing distance from the impact point (e.g., Ferrière et al. [2008] and references therein; Holm‐Alwmark et al. 2017).

While the pressure range in which shock microstructures in quartz form is relatively well constrained (e.g., Stöffler and Langenhorst 1994; Grieve et al. 1996; Huffman and Reimold 1996; and references therein), shock metamorphic features in feldspar have not been investigated in such detail. This is due not only to feldspar being often completely altered but also due to its complex atomic arrangement, mostly determined by the different valence of Na and K with respect to Ca, which is responsible for the biaxial nature of feldspar and the presence of monoclinic and triclinic symmetries, depending on the composition. The fact that the shock pressure necessary to amorphize plagioclase is lower for Ca‐rich varieties than for Na‐rich varieties is relatively well known (e.g., Kubo et al. 2010; Fritz et al. 2011). However, planar fractures (PFs), PDFs, and possibly the occurrence of alternating amorphous microtwins (Dworak 1969; Pickersgill et al. 2015) have been rarely described in shocked feldspar in shock recovery experiments, and in naturally shocked samples in meteorites and in terrestrial impactites (e.g., Stöffler 1967; Ostertag 1983; Pittarello et al. 2013). Based on the occurrence of shock microstructures in plagioclase reported in the literature, the presence of PDFs seems to be limited to intermediate to albitic terms (no PDFs in An_63_, Gibbons and Ahrens 1977, but present in An_<54_, e.g., Stöffler 1967; Ostertag 1983; White 1993; Langenhorst et al. 1995; Kayama et al. 2009; Pittarello et al. 2013; Jaret et al. 2014). The occurrence and distribution of shock microstructures in quartz and plagioclase within a given sample appear heterogeneous and randomly distributed at the thin section scale.

Together with PDFs in quartz, shatter cones represent confirming evidence of shock wave passage across rocks (e.g., French and Koeberl 2010). Their formation process is still debated, despite the fact that shatter cones have been reproduced in shock experiments (e.g., Baratoux and Reimold 2016; Osinski and Ferrière 2016; and references therein). Shatter cones form in all possible lithologies, disregarding their mineralogical composition or grain size; they occur in the target rock, commonly in the central uplift, but also as clasts in impact breccias. Even though the hypothesis that the apex of shatter cones point to the impact center has been disproved (e.g., Wieland et al. 2006; Osinski and Ferrière 2016), the striations on shatter cone surfaces are generally considered as traces of the local scattering of shock waves, triggered by a heterogeneity in the target rock (e.g., Baratoux and Melosh 2003; Dawson 2009; Baratoux and Reimold 2016; Osinski and Ferrière 2016). However, alternative formation models for shatter cones do not require the presence of heterogeneities in the target rock, but are based on the speed of shock wave propagation (e.g., Sagy et al. 2002, 2004; Kenkmann et al. 2016).

Here, we present the first investigation of shock‐induced planar microstructures in quartz and feldspar in thin sections cut along specific orientations, to determine whether a preferred distribution in frequency and orientation exists and which information on the local (sample‐scale) shock wave propagation direction this distribution can provide. A somewhat similar approach to ours, but limited to PDFs in quartz, was tested by Poelchau et al. (2014), and more recently applied to feather features (FFs) in quartz by Ebert et al. (2019), in the attempt to detect a deviatoric stress contribution in the shock wave. Both studies show some limitations that are considered and discussed in this work.

## METHODS

**Fig. 1:**
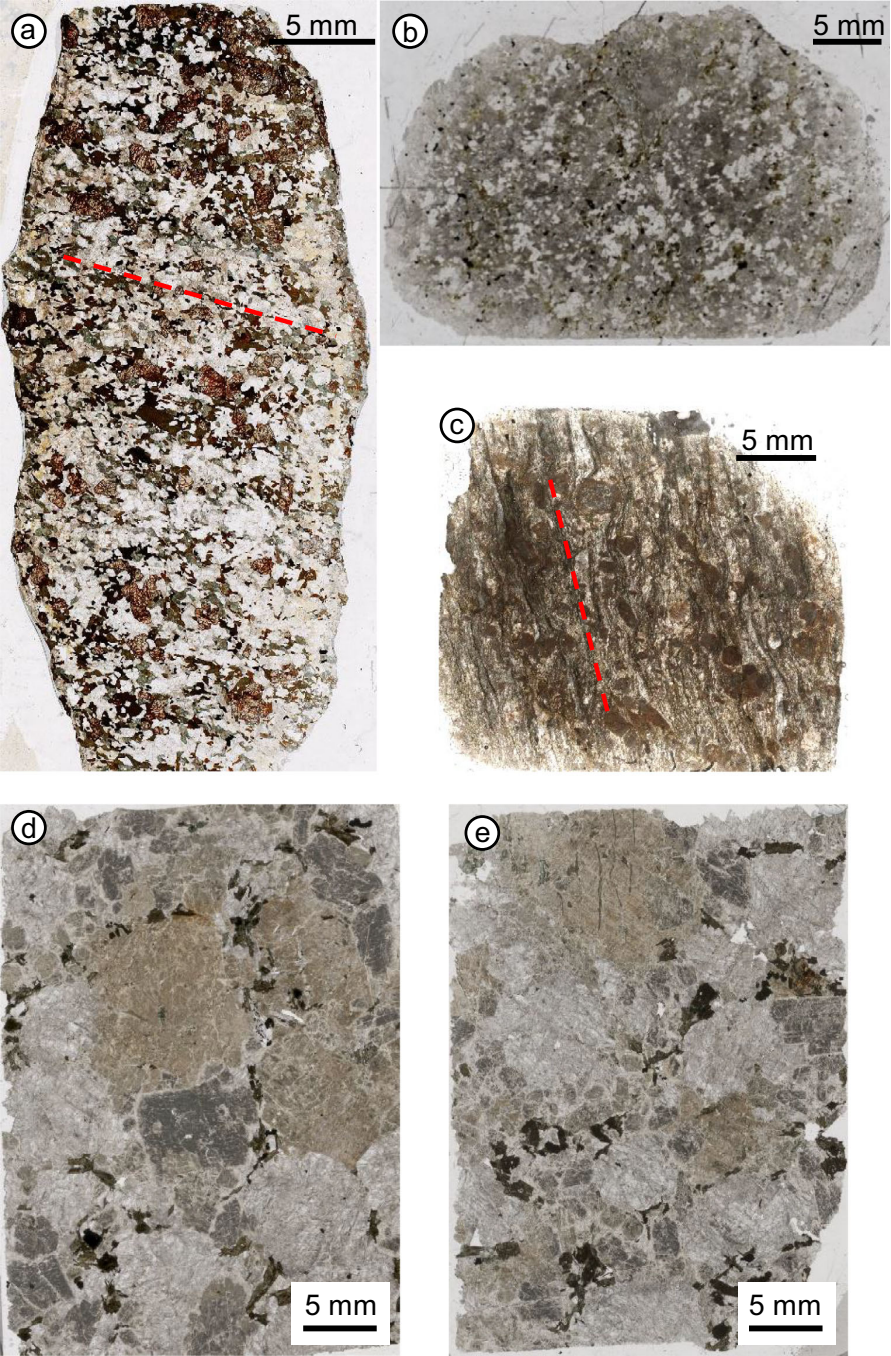
Selection of thin sections investigated in this study. a) Sample WMM‐102A‐64C1 from the Manicouagan impact structure, thin section cut normal to the striated surface and normal to shatter cone striations. The weak foliation is marked with a red dashed line. b) Sample CHA09‐12‐01 from the Charlevoix impact structure. c) Sample VN3 from the Keurusselkä impact structure. Note the strong foliation, marked with a red dashed line. d) Sample 188R2_11‐13.5 (986.19 mbsf) from the Chicxulub drill core. Thin section cut parallel to the drill core axis (V). e) Same sample as in (d), but the thin section was cut normal to the drill core axis (H). Note the coarse grain size. (Color figure can be viewed at wileyonlinelibrary.com.)

As pre‐impact tectonic deformation can play a role in imposing a preferred crystallographic orientation of minerals, target rocks recording three different stages of pre‐impact tectonic deformation, from undeformed to strongly foliated, were considered. The selected samples are from Manicouagan (Canada; weakly foliated; e.g., Dressler 1990; Spray et al. 2010; Fig. 1a), Charlevoix (Canada; no foliation, e.g., Robertson 1975; Trepmann and Spray 2005; Fig. 1b), and Keurusselkä (Finland, strongly foliated; e.g., Ferrière et al. 2010; Fig. 1c) impact structures. Petrographic thin sections were cut roughly normal to shatter cone surface and striation, considering their average orientation on the sample scale.

In addition, five granite samples from the joint IODP‐ICDP Chicxulub Expedition 364 drill core (e.g., Morgan et al. 2016) were selected at different depths: 132R1_54‐57 (838.76 m below sea floor, mbsf), 164R2_47‐52 (919.52 mbsf), 188R2_11‐13.5 (986.19 mbsf), 212R1_129‐131.5 (1056.01 mbsf), and 224R1_61‐63.5 (1091.39 mbsf). For these samples, the drill core axis was used as reference, although the samples were not oriented, which means that the only known parameters are the axis of the drill core and the upper versus lower surface orientation. Petrographic thin sections were cut parallel to the drill core axis (marked as V for vertical) and normal to the drill core axis (marked as H for horizontal) from only three of these five samples (132R1_54‐57, 188R2_11‐13.5, and 212R1_129‐131.5), considered as representative of the investigated drill core sections (Figs. 1d and 1e). One thin section from each of the remaining two samples were also investigated for shock features and considered for this work, but their contribution is only in one cut direction, as it was not possible to obtain a second thin section cut along another orientation from the same sample.

The orientation of planar microstructures, such as PDFs in quartz, PFs, PDFs, and (likely shock‐induced) microtwins in feldspar, as well as the orientation of the *c*‐axis in quartz grains containing PDFs were determined using the universal‐stage (U‐stage), following the method described in Passchier and Trouw (1996) and Langenhorst (2002), and plotted with the program Stereo32 (formerly supported by K. Roeller and C. Trepmann of Ruhr‐Universität Bochum, Germany). Although the U‐stage allows the investigation of planar microstructures in 3‐D over a statistically meaningful number of grains, the method presents some intrinsic limitations. The geometry of the U‐stage limits the area of the thin section that can be investigated to its central portion (i.e., only approximately three‐fifths of a standard rectangular thin section). This reduces the number of grains that can be measured, especially in the case of coarse‐grained samples or when shocked grains are rare. It should be emphasized that the geometry of the U‐stage also limits the angle of planar microstructure that can be measured to a central cone of visible orientations, corresponding to low angle pole distributions (maximum about 50°–55°).

## Results

### Petrographic Description

**Fig. 2:**
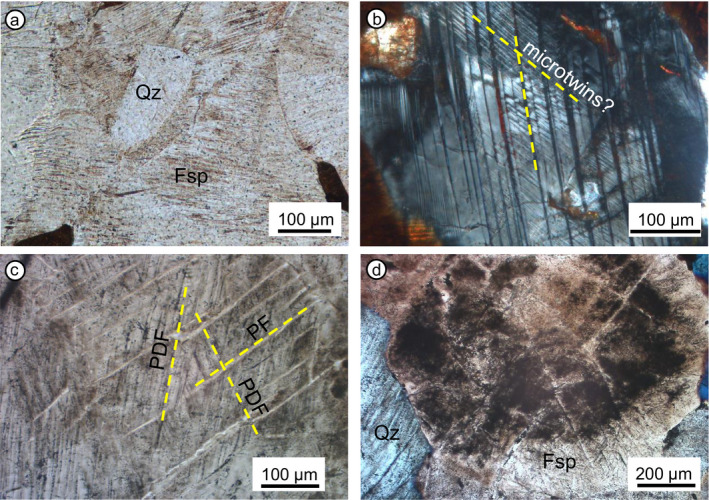
Shock‐related microstructures observed in the investigated samples. a) Almost unshocked quartz grain (at higher resolution, it shows one to two PDF sets) surrounded by plagioclase containing abundant planar microstructures. Sample WMM‐102A‐64C1. Plane polarized light microphotograph. b) Multiple sets of twins with reduced birefringence or completely amorphous and possible PDFs in plagioclase. Sample WMM‐102A‐64C1. Thin section between crossed polarizers. c) Multiple PDFs sets in quartz. Sample 188R2_11‐13.5. Between crossed polarizers. d) Set of planar microstructures in the Ab‐rich rim of a plagioclase grain. Note the extensive alteration (sericitization) of the core of the grain, more An‐rich than the rim. Sample 132R1_54‐57, crossed polarizers. (Color figure can be viewed at wileyonlinelibrary.com.)

The shatter cone sample from Manicouagan, WMM‐102A‐64C1, consists of a weakly foliated, garnet‐bearing metagranite (Fig. 1a). Foliation is defined by elongated ribbons of biotite, partly replaced by chlorite, generally departing from intensely fractured garnet crystals. Apart from irregular fracturing, which is non‐diagnostic for shock, shock metamorphic features are mostly concentrated in oligoclase and in a few quartz grains. Planar elements in oligoclase very likely related to shock include extensive microtwinning and possible PDFs (Pittarello, personal communication), forming a dense network of planar microstructures (Figs. 2a and 2b). Locally, the possible PDFs develop only in individual synthetic twins or only one individual appears isotropic, as already observed by Dressler (1990). Shock features in quartz include PDFs, generally just one set, and oriented parallel to $\left\{10\overset{-}{1}3\right\}$. Our observations are consistent with the shock microstructures found in plagioclase in the target rock by Dworak (1969), Dressler (1990), and White (1993).

The sample from Charlevoix, CHA09‐12‐01, consists of an undeformed granitoid, mainly composed of quartz, feldspar, hornblende, and opaque minerals (Fig. 1b). Quartz is shocked and exhibits up to three PDF sets per grain (as seen under the U‐stage), mainly oriented parallel to the $\left\{10\bar{1}3\right\}$ and (0001). Feldspar crystals, generally weathered, show PFs and microtwins or possible PDFs (difficult to determine just optically).

The sample from Keurusselkä, VN3, consists of a strongly foliated orthogneiss, with elongated quartz ribbons, subrounded sericitized feldspar, and partially chloritized biotite layers (Fig. 1c), as described in Ferrière et al. (2010). Quartz exhibits core and mantle structure with tectonic, extensive dynamic recrystallization. Quartz grains larger than 50 μm contain multiple sets of decorated PDFs, up to four sets (as seen under the U‐stage), mainly oriented parallel to $\left\{10\bar{1}3\right\}$ and $\left\{10\bar{1}4\right\}$, but no basal PDFs were measured; PFs are rare (Ferrière et al. 2010). A few feldspar grains are present in the groundmass and are characterized by microtwinning.

The selected Chicxulub samples consist of coarse‐grained (basement) granite, forming part of large blocks displaced in the peak ring of the Chicxulub impact structure. These granites mainly consist of K‐feldspar (orthoclase); partially sericitized plagioclase; and quartz; and, to a lesser extent, extensively chloritized biotite, muscovite, apatite, titanite, epidote, zircon, opaque minerals, and other accessory minerals. The five samples share some common features, such as their coarse grain size (mm size) and extensive alteration (i.e., chloritization of biotite and sericitization of plagioclase; Figs. 1d and 1e). Quartz grains are highly shocked; most of them, if not all, exhibit mostly decorated PDFs (Fig. 2c), up to five sets per grain (as seen under the U‐stage), PFs, locally FFs, and strong undulose extinction. Plagioclase presents planar elements like microtwins, PFs, and very likely PDFs in a somewhat lesser abundance than quartz, but plagioclase is preferentially altered and alteration might have obliterated shock effects. Locally, planar microstructures in plagioclase are concentrated at the rim of the grains, which have lower An contents than the cores of magmatic zoned plagioclase grains (Fig. 2d). Detailed work on the shock metamorphic features in quartz, and in particular on the shock effects recorded in other minerals contained in these samples from the IODP‐ICDP Chicxulub Expedition 364 drill core is the subject of an another study (Feignon, personal communication).

### Orientation Data

**Fig. 3:**
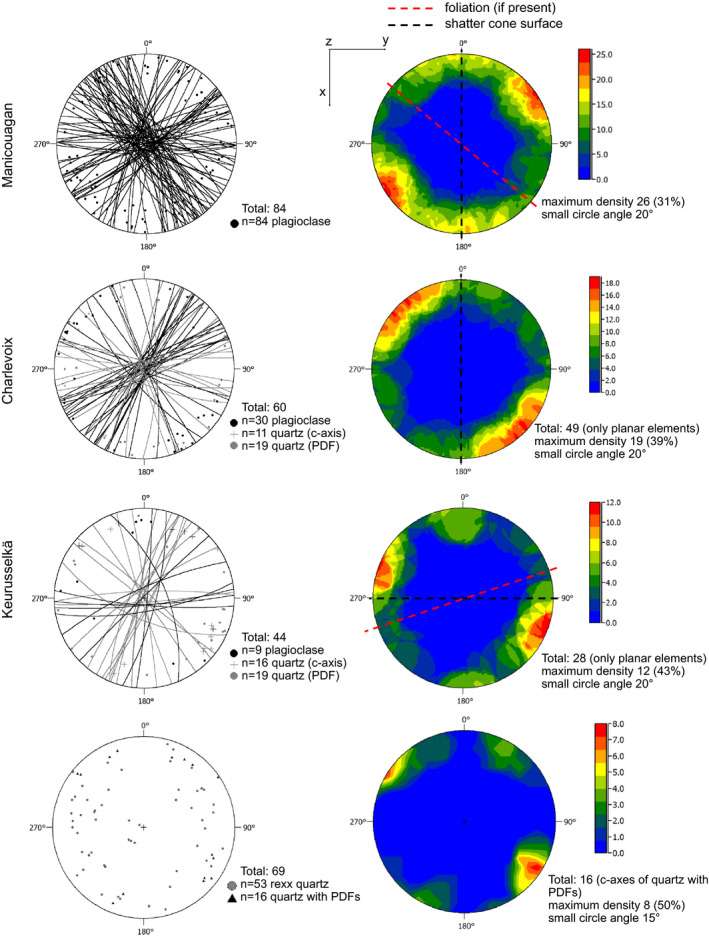
Stereographic projections and contour plots of the planar microstructures in quartz and plagioclase from the studied shatter cone samples, as well as the orientation of quartz *c*‐axes in the case of the Keurusselkä sample. All the thin sections were cut normal to the striated surface of shatter cones and normal to striations. The reference system is indicated in the figure, with *x* vertical, *y* horizontal, and *z* normal to the observer. Equal angle projection, lower hemisphere. In the contour plots, bins of 20° angles are considered. The frequency distribution is also indicated, to allow a comparison of the frequency of similar orientations. The orientation of the striated surface of shatter cones and that of the foliation, whenever present, are marked in the contour plots. Note that for all three considered samples, the maximum frequency of planar microstructure orientations is at an angle between 30° and 45° from the average orientation of the shatter cone surface. For the sample from the Keurusselkä impact structure, where a strong foliation is present, the orientations of quartz *c*‐axes are also plotted. Note that the dynamically recrystallized quartz grains (rexx quartz) have a random distribution, whereas quartz grains containing PDFs define a preferred orientation that is consistent with that shown by the planar microstructures, even though the number of considered grains is too low for extended consideration. (Color figure can be viewed at wileyonlinelibrary.com.)

In Fig. 3, the stereographic projection and the contour plots of the orientations of planar microstructures in quartz and feldspar from the shatter cone samples are shown. Whenever a foliation was obvious, its orientation is marked in the plot. For all the samples, the projection of the striated surface orientation with respect to the thin section is also shown. In the case of the Manicouagan sample, all measurements are planar microstructures in plagioclase, because only a few quartz grains were present (i.e., three grains, each with one obvious set of PDFs oriented along $\left\{10\overset{-}{1}3\right\}$, which is statistically meaningless). The crystallographic orientation of plagioclase grains that contain planar microstructures was not evaluated, due to the complexity of such measurement with the U‐stage (e.g., Stöffler 1967), the presence of a pre‐shock foliation, and the time and costs required for electron backscatter mapping of the whole thin section. In the case of the Charlevoix sample, planar microstructures in plagioclase and PDFs in quartz were measured. Although no foliation is obvious at the thin section scale in this sample, a slight preferred orientation of the planes in the NE–SW quadrants of the stereographic projection is observed for feldspar microstructures. The orientations of the *c*‐axes of quartz grains containing PDFs show a random orientation on the surface normal to z (i.e., the thin section surface). In the case of the Keurusselkä sample, only features in porphyroclasts were measured, because the dynamically recrystallized quartz matrix has grain size that is too fine to allow the detection of possible shock features with the optical microscope. However, quartz *c*‐axis orientation of such porphyroclasts still defines a preferred direction, likely due to porphyroclasts rotation (e.g., Mancktelow et al. 2002). For all analyzed samples, no relation between the tectonic deformation degree and the presence of preferred orientation of shock‐related planar microstructures was observed, that is, the observed preferred orientation does not reflect pre‐impact tectonic deformation. All samples exhibit a maximum in the orientation distribution of the poles of planar microstructures, which has angles in the range 30°–45° with respect to the average orientation of the striated shatter cone surface at the thin section scale.

**Fig. 4:**
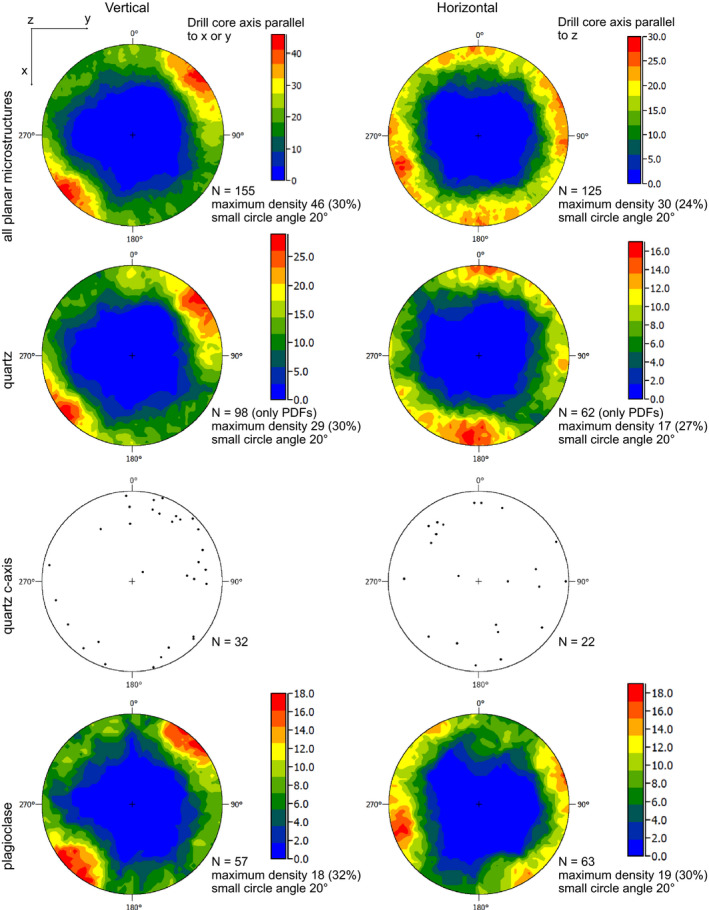
Contour plots from the stereographic projections of the planar microstructures in quartz and plagioclase and *c*‐axes of quartz grains containing PDFs in the samples from the Chicxulub drill core. Equal angle projection, lower hemisphere. The reference system is indicated in the figure, as well as the orientation of the plots with respect to the drill core axis. Bins of 20° angles are considered. Comparison between preferred orientations of planar microstructures in thin section cut parallel to the drill core axis (Vertical) and normal to the drill core axis (Horizontal), as indicated in Table 1. Summary data, for quartz and plagioclase, respectively, are presented. Individual plots for the considered samples are provided in the supporting information. Note the pattern defined by maximum frequency of orientations in the “vertical” sections and the higher frequency of planar microstructures in the “vertical” sections than in the “horizontal” sections. (Color figure can be viewed at wileyonlinelibrary.com.)

In Fig. 4, the stereographic projections obtained from the Chicxulub samples are distinguished between H and V thin sections. Due to the limited number of grains that could be measured per thin section, the contribution of all investigated thin sections was plotted for each direction, disregarding the possible deviation from ideal verticality or horizontality of the cut with respect to the axis of the drill core, that is, angle of the surface with respect to the drill core axis <90° for the horizontal thin sections. The selected bin (i.e., small circle of 20°) for orientations was applied to both H and V thin sections, to highlight also the difference in the absolute frequency (see also Table 1). Planar microstructures considered as a whole (i.e., quartz + feldspar contribution) in H thin sections are less abundant and have pole orientations more spread along the low angle domains than those in V thin sections. The latter shows a clear pattern, with a well‐defined maximum at approximately 30° from the *x*‐axis (convention, *x* vertical, *y* horizontal, and *z* normal to the observation plane). Considering the orientation of planar microstructures, we observe that, in plagioclase, the orientation pattern in the V thin sections is very strong, but the number of detected microstructures is less abundant in absolute values than in the H thin sections. In the case of quartz, the difference in the preferred orientations of PDF poles between H and V sections is less obvious than in plagioclase, but the number of measured features is much higher in V thin sections with respect to H thin sections (Fig. 4). In addition, the orientation distribution of the c‐axes of quartz grains containing PDFs is clearly random in the H thin sections, but shows a weak preferred orientation in V thin sections. The maximum observed in the orientations of planar microstructures in the V sections has a similar shape as that obtained for the shatter cone thin sections.

**Table 1:** Number of planar microstructures in quartz and feldspar and quartz *c*‐axis orientations per thin section considered in this work. The orientation of the thin section with respect to a reference system (striated surface of shatter cones or drill core axis) is provided: V for vertical, which means normal to the striated surface and to striations in shatter cones and parallel to the drill core for Chicxulub samples, and H for horizontal, which means normal to the drill core axis.

	Thin section	Orientation	Quartz		Feldspar	Total
			*c*‐axis	PDFs		
Shatter cones	Charlevoix (undeformed)	V	11	19	30	60
	Manicouagan (weak foliation)	V	—		84	84
	Keurusselkä (strong foliation)	V	16	19	9	44
Chicxulub	132R1_54*	H	6	22	23	51
	132R1_54*	V	10	24	26	60
	164R2_47	H?	5	13	21	39
	164R2_47	V	11	23	25	59
	188R2_11*	H	6	15	16	37
	188R2_11*	V	9	41	7	57
	212R1_129	V	6	12	8	26
	224R1_61*	H	10	25	24	59
	224R1_61*	V	13	33	24	70
	Orientations considered from *thin sections	H	22	62	63	147
	Orientations considered from *thin sections	V	32	98	57	187

## Discussion

The formation mechanism of shatter cones is still debated and it has been demonstrated that the apex or striation orientation has no relation with the center of the crater or shock wave propagation direction, as shatter cones with multiple orientations have been described within a given sample (e.g., Wieland et al. 2006; Osinski and Ferrière 2016; Baratoux and Reimold 2016; and references therein). However, on the local scale, immediately below the striated surface, we can assume that the shock wave propagated roughly homogeneously and roughly parallel to the striation direction, spreading radially. The local shock wave scattering can have been triggered by the presence of heterogeneities (e.g., Baratoux and Melosh 2003; Dawson 2009) or simply by bifurcations (e.g., Sagy et al. 2002, 2004; Kenkmann et al. 2016). This assumption is supported by the fact that all three samples of shatter cones show a clear preferred orientation of the poles of shock‐induced planar microstructures in quartz and feldspar with respect to the striated surface of the shatter cones. In addition, this preferred orientation is not related to, and thus not controlled by, the tectonic deformation degree experienced by the samples. Due to limited access to the samples from which the investigated thin sections were prepared, it was not possible to verify the presence of this preferred orientation along other sections, cut normal and parallel to the striation. The angle between the preferred orientation of the poles of the planar microstructures and the average local striated surface in the thin section is in the range 30–45°. This relatively large range is likely due to the conical shape of the striated surface, also at the size of the thin section.

The shock pulse duration is too short with respect to tectonic deformation to induce changes in the crystallographic preferred orientation of minerals (e.g., Vernooij et al. 2006; Trepmann 2008). However, we can assume that grains with crystallographic orientation favorably oriented with respect to the shock wave propagation direction will preferentially develop shock‐induced planar microstructures. This would explain the variety of frequency of such planar microstructures within a given thin section (i.e., at thin section scale). This mechanism is likely responsible also for the formation of the observed maxima in the orientation of planar microstructures in quartz and feldspar (Fig. 3). The relatively broad maxima for the preferred orientations in the case of the Chicxulub samples likely reflect variations in the spatial orientations of the granite blocks within the peak ring.

Due to the U‐stage geometry restrictions, planar microstructures with high angles with respect to the microscope stage are more easily detected than those with low angles (e.g., Holm et al. 2012). For this reason, all plots exhibit roughly similar stereographic projection of shock planar microstructures, with the poles concentrated along the margin of the stereographic projections. This confirms that a certain bias induced by the methodology cannot be ignored. Nevertheless, contour frequency plots highlight a significant difference between normal versus parallel cut directions (Fig. 4). Notably, the contour frequency plots for vertical thin sections from the Chicxulub drill core are similar in shape to those obtained from the thin sections cut normal to the striated surface of the shatter cones. This trend is observed for all samples, except in one case, where a “vertical” trend is obtained for both thin sections (e.g., sample 164R2_47‐52, Table 1 and supporting information), likely due to the rotation of the target rock block during the modification stage of impact cratering or to the tilt of the sample during drilling.

An earlier attempt to constrain the compression direction of shock waves by investigating with the U‐stage, the preferred orientation of PDFs in quartz (Poelchau et al. 2014) was limited to one thin section. Considering the above‐mentioned limitations induced by the U‐stage, we recommend that the preferred orientation of PDFs should be evaluated in thin sections cut along additional orientations. A more recent attempt applied to FFs in quartz from the Expedition 364 Chicxulub drill core (Ebert et al. 2019), based on the assumption that FFs indicate the shear sense along the PFs from which they originate, also suffers from some limitations. In this case, the major issue is the extrapolation on the regional scale of the blocks tilting in the peak ring, without having any control on whether or not the thin sections were cut vertically or horizontally with respect to the drill core axis.

Although the general idea to use shock‐induced microstructures in quartz and feldspar to derive the local (sample scale) shock wave propagation direction looks promising, the complexity of processes occurring during impact cratering, in particular during the modification stage, hampers any further conclusion on reconstruction of the shock wave propagation direction. However, with dedicated experiments, a shock direction calibration for the very local (microscopic) scale might be determined, and possibly extrapolated on analysis of a statistically meaningful number of samples. The presence of test rocks with a strong pre‐impact tectonic deformation recorded in quartz and feldspar would allow the investigation of the interaction between the shock wave propagation and the crystallographic preferred orientation in the target, to enhance or reduce the intensity of the shock effects.

## Conclusions

We show in this study that the orientations of planar microstructures related to shock in quartz and feldspar, including PDFs in quartz, and PFs, PDFs, and microtwins in feldspar, considered as a whole, define a pattern, which is different depending on the considered sections cut of the samples. This implies that the orientation distribution of such planar microstructures is not random in 3‐D, but is likely controlled by the relation between the local shock wave and the crystallographic orientation of individual crystals.

In shatter cone samples, planar microstructures investigated in a section cut normal to the striated surface and the striation direction show a strong preferred orientation, with an arrangement of most of the shock‐related planar microstructures in quartz and in feldspar almost subparallel to the striated surface. In samples from the Chicxulub Expedition 364 drill core, the preferred orientation of planar microstructures is less obvious. However, most of the sections cut subparallel to the drill core axis show similar distribution of planar microstructures in quartz and feldspar as in the investigated shatter cone samples. Therefore, we can assume that these microstructures do not form randomly, but that their orientation is somehow controlled by the local shock wave direction. However, due to the uncertainty on the original orientation of the blocks in the Chicxulub peak ring, an extrapolation of this conclusion to the regional (crater) scale is not possible. Additional studies, possibly investigating experimentally shocked samples, where the orientation of the shock wave is well constrained, will further support the present observations. The final aim would be to derive a calibration, which will possibly allow us to determine statistically the shock wave direction at the crater scale.

## Editorial Handling

Dr. Jeffrey Plescia

## Supporting information


**Fig. S1.** Individual contour plots for the investigated samples from the Chicxulub drill core. Planar microstructures and c‐axis orientations of quartz grains that contain PDFs are summarized, as in Table 1. Bins of 20° were considered. The frequency of orientations within a bin is indicated in percent.
