# Supplementary material for: Preferred orientation distribution of shock‐induced planar microstructures in quartz and feldspar
Source: Meteorit Planet Sci. 2020 Jun 4;55(5):1082–92. doi: 10.1111/maps.13490 (PMC7508181; doi:10.1111/maps.13490)
Supplement: Supplementary file 1 — Fig. S1. Individual contour plots for the investigated samples from the Chicxulub drill core. Planar microstructures and c‐axis orientations of quartz grains that contain PDFs are summarized, as in Table 1. Bins of 20° were considered. The frequency of orientations within a bin is indicated in percent. [file MAPS-55-1082-s001.pdf]

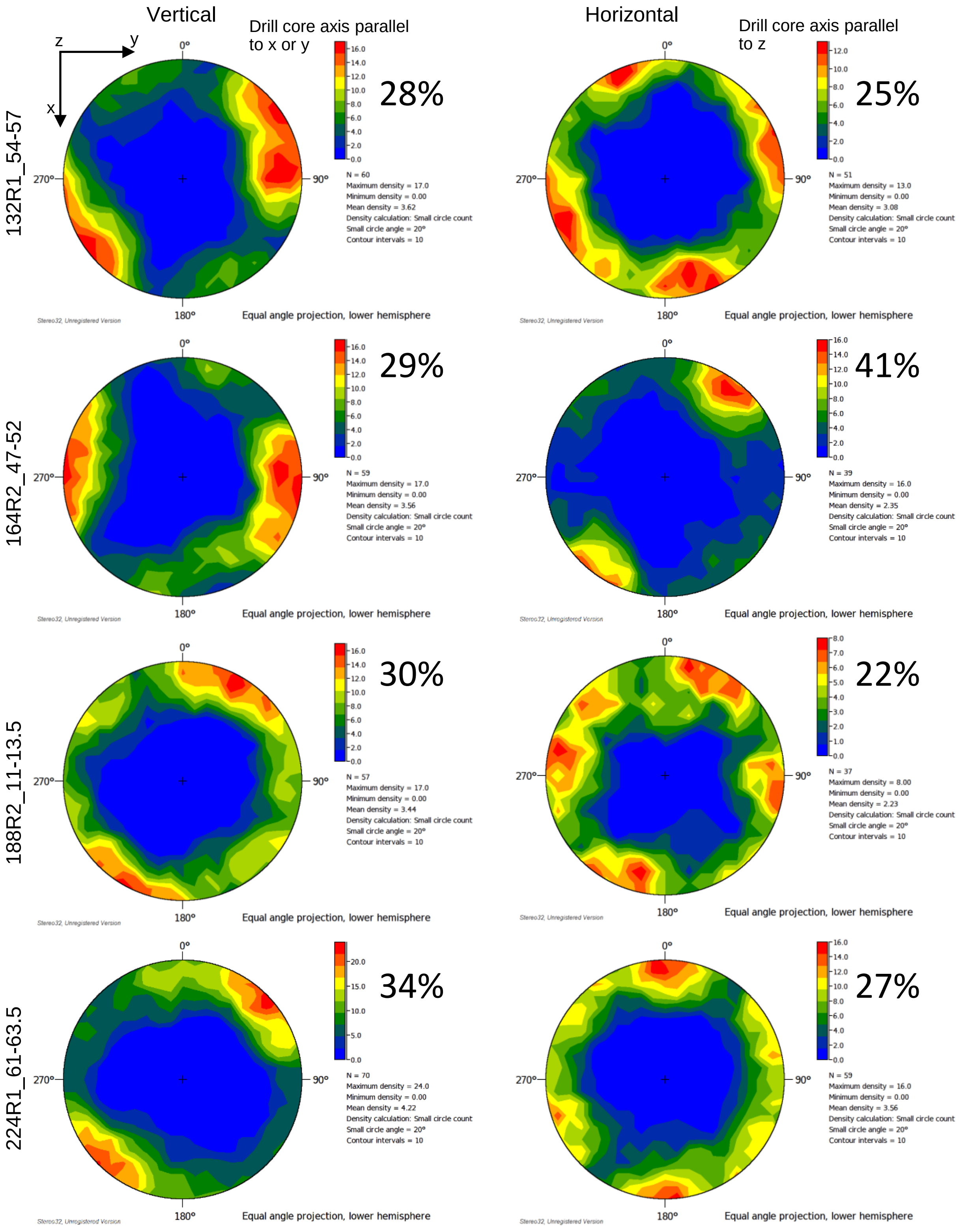

Suppl. Mat. Individual contour plots for the investigated samples from the Chicxulub drill core. Planar microstructures and c-axis orientations of quartz grains that contain PDFs are summarized, as in Table 1. Bins of 20° were considered. The frequency of orientations within a bin is indicated in percent.
